# Supplementary material for: Coherent control in the extreme ultraviolet and attosecond regime by synchrotron radiation
Source: Nat Commun. 2019 Nov 1;10:4988. doi: 10.1038/s41467-019-12978-w (PMC6825198; doi:10.1038/s41467-019-12978-w)
Supplement: Supplementary file 1 — Supplementary Information [file 41467_2019_12978_MOESM1_ESM.pdf]

Coherent control in the extreme ultraviolet and attosecond regime by synchrotron radiation

Hikosaka et al.

Supplementary Information

Supplementary note 1

Supplementary figure 1 presents the time spectra for the contributions from 3p and 7p&8p, as well as the re-plots of the time spectra shown in Fig. 3 of the manuscript. Fluorescence lines from 7p and 8p could not be resolved with the band-pass filter used, both contributions are included in the spectrum in supplementary figure 1(b). The statistics (5% error) of the spectrum for 3p is worse than those (1-2% error) of other spectra, owing to low count rate and large background contribution (which was subtracted already in the spectrum).

The time spectra in supplementary figure 1, reflecting the populations of the corresponding Rydberg states, oscillate with individual frequencies. The population ρ of a Rydberg state at a time delay τ between the light wave packet pair is given by

$$\rho \propto (1 + \cos \omega\tau) \ , \qquad (1)$$

where ω is the transition frequency from the ground state to the Rydberg state[1-3]. In supplementary figures 1(b)-(f), the best fits to the experimental spectra (dots) with the sinusoidal function (supplementary eq. 1) convoluted by Gaussian are shown by solid lines. These spectra indicate the contributions included in the total fluorescence spectrum. The general trend in the total fluorescence spectrum can be well reproduced by summing the fitting spectra in supplementary figure 1(b)-(f), after multiplying a factor of 0.5 and 2 to the spectra for 3p and 7p&8p, respectively. The sum spectrum is presented in supplementary figure 1(a) as a red solid curve, where a constant background of 0.62 was added in order to reproduce the fringe contrast in the total fluorescence spectrum. This fact implies that the total fluorescence includes the corresponding magnitude of background.

The reasonable reproduction of the spectral feature in the total fluorescence spectrum by the sum spectrum implies that the contributions from 4p-8p are comparable in the total fluorescence, while that from 3p is about half. These contributions are determined by the undulator spectrum and the state-dependent branching ratios for the fluorescence decays. The ramp-down seen around 2500 as in the total fluorescence spectrum is insufficiently reproduced in the sum spectrum, suggesting non-negligible contributions from higher-n states.

References

[1] Amand, T., Blanchet, V., Girard, B. & Marie, X. Coherent Control in Atoms, Molecules and Solids. in *Femtosecond Laser Pulses. Advanced Texts in Physics* (Springer, 2005)

[2] Ohmori, K., Wave-Packet and Coherent Control Dynamics, *Ann. Rev. Phys. Chem.* **60,** 487–511 (2009).

[3] Ohmori, K., Sato, Y., Nikitin, E. E. & Rice S. A. High-precision molecular wave-packet interferometry with HgAr dimers. *Phys. Rev. Lett.* **91,** 243003 (2003).

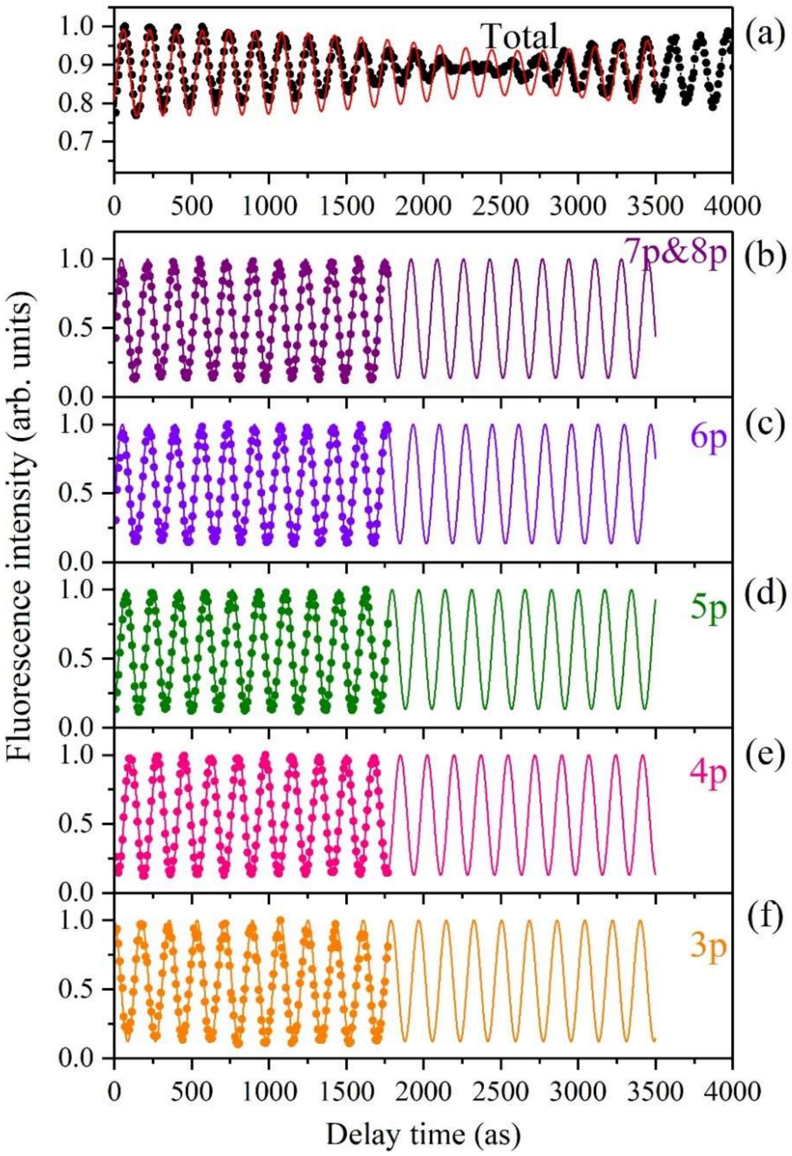

Supplementary figure 1. Intensities of fluorescent photons from He. Fluorescence intensities were measured as a function of the time delay between the light wave packets: (a) total fluorescence in the visible and ultraviolet regions and (b)-(f) contributions from the p-type Rydberg states of n=3-8. The black/dots spectrum in (a) and the spectra in (c)-(e) are the replots from Fig. 3 of the manuscript. The fluorescence intensities were normalized by the intensities of the undulator radiation and then multiplied so that the maxima become unity. In (b)-(f), while the experimental values (dots) for the individual Rydberg states were measured until a time delay of 1770 as, the best fits to the experimental values with a sinusoidal function convoluted by Gaussian are depicted with solid lines continuing up to a time delay of 3500 as. The sum of the fitting spectra in (b)-(f), after multiplying a factor of 0.5 and 2 to the spectra for 3p and 7p&8p, respectively, is shown in (a) as a red sold curve.
